# Defining lactation outcomes, milk composition, and breastfeeding safety for women with chronic kidney disease: protocol for a prospective observational study

**DOI:** 10.1186/s13006-026-00821-0

**Published:** 2026-02-21

**Authors:** Anna Sadovnikova, Masani Coley, Lanise Powell, Adrienne Hoyt-Austin, Herman L. Hedriana, Kara M. Kuhn-Riordon, Russell C. Hovey, Carolyn M. Slupsky, Flora Zhang, Nandakishor Kapa

**Affiliations:** 1 Division of Breastfeeding and Lactation Medicine, Department of Pediatrics, University of Rochester, Rochester, USA; 2 University of California, Davis School of Medicine, Sacramento, USA; 3 Department of Pediatrics, UC Davis Health, Sacramento, USA; 4 Division of Maternal-Fetal Medicine, Department of Obstetrics and Gynecology, UC Davis Health, Sacramento, USA; 5 Department of Animal Science, University of California, Davis, USA; 6 Department of Nutritional Sciences, University of California, Davis, USA; 7 Department of Internal Medicine, Division of Nephrology, UC Davis Health, Sacramento, USA

**Keywords:** Lactation, Protocol, Chronic kidney disease, Human milk collection, Milk composition, Kidney transplant, Dialysis, Hemodialysis, Breastfeeding, Cohort

## Abstract

**Background:**

Among persons of childbearing age, advanced chronic kidney disease (stage 3–5) reportedly affects approximately 1 in 150 individuals. Birth rates compared to the general population are lower in kidney transplant recipients and in individuals undergoing dialysis, estimated at approximately 1:10 and 1:100, respectively. Despite these challenges, the incidence of pregnancy among those with chronic kidney disease is increasing, and birthing parents with advanced chronic kidney disease, including those undergoing dialysis or with a kidney transplantation, express a strong desire to breastfeed prenatally and initiate breastfeeding after childbirth. However, the factors influencing breastfeeding outcomes and their milk composition in birthing parents with kidney disease are not well understood. At present, no clinical practice guidelines exist to specifically address lactation management for birthing parents with kidney disease, leaving a gap in support and care for this population.

**Aims:**

Our primary aims are to describe lactation outcomes and define milk composition for birthing parents with advanced chronic kidney disease, post-kidney transplantation, or receiving dialysis therapy. Our secondary aims are to describe the breastfeeding experience, including attitudes, self-efficacy, and challenges, and evaluate infant health outcomes including developmental and medical history.

**Methods:**

We are conducting a prospective observational cohort study in California, using mixed methods to examine lactation outcomes, breastfeeding experience, parent-infant health, and milk composition among participants (*N* ≈ 10) with chronic kidney disease, kidney transplantation or dialysis. Participants will complete surveys, oral interviews, breastfeeding telemedicine consultations, 24-hour infant test-weighing, and milk sample collection. Control participants without kidney disease and with either normal (600–1035 mL) or low (< 600 mL) milk supply will be recruited. Milk composition (macronutrients, metabolome, electrolytes, cytokines, and immunoglobulins) will be compared. We will use descriptive statistics to summarize participant characteristics, and inductive thematic analysis to generate contextual insight into lactation patterns and birthing parent experiences.

**Discussion:**

Outcomes of this study will guide practice recommendations for the multidisciplinary care team supporting birthing parents with advanced chronic kidney disease, kidney transplantation or undergoing dialysis, ensuring breastfeeding safety and appropriate support.

**Supplementary Information:**

The online version contains supplementary material available at 10.1186/s13006-026-00821-0.

## Background

Breastfeeding offers significant health benefits for birthing parents and their infants, including reduced risk of hypertension, cardiovascular disease, and Type 2 diabetes [1–3]. Breastfeeding is a critical intervention for birthing parents with chronic kidney disease (CKD), who often have multiple comorbidities, such as hypertension, obesity, and diabetes [4, 5]. For infants, breast milk, compared to formula, significantly reduces the risk of respiratory and gastrointestinal infections, including necrotizing enterocolitis [6, 7]. The effect of breast milk on reducing infection risk is particularly relevant for infants of birthing parents with CKD, who are more likely to be born prematurely and require care in the neonatal intensive care unit (NICU) [8–10]. These evidence-based benefits highlight the importance of bridging knowledge gaps in lactation management for birthing parents with CKD to improve lactation support for this population.

Given the rising incidence of successful pregnancy with advanced CKD as well as renal transplantation, it is essential to develop evidence-based recommendations for lactation support, including strategies to overcome breastfeeding barriers and optimize maternal and infant health outcomes for this population [5, 9, 11]. Despite a growing number of publications about pregnancy management, complications, and outcomes, there are few studies in which the lactation outcomes and breastfeeding experiences for this population are described [11–13]. In one study, breastfeeding women with CKD, on dialysis, or with a history of a kidney transplantation reported conflicting medical advice regarding breastfeeding safety and medication compatibility [11]. Some investigators have reported concerns about the milk composition and therefore, breastfeeding safety, for the infants of breastfeeding women receiving hemodialysis treatment [12, 13]. However, these case studies did not report lactation complications, breastfeeding history, or 24-hour milk volumes for either the patient or control groups, which may help explain some of the observed protein and electrolyte abnormalities [14–17]. Breastfeeding is recommended and encouraged for birthing parents with altered milk composition, as may occur in diabetes, obesity, or who take medications for chronic conditions, including for kidney disease [5, 18–20]. Thus, it is imperative to study breastfeeding outcomes, infant health outcomes, and milk composition for birthing parents with kidney disease to inform evidence-based recommendations.

Herein, we present a protocol for a prospective observational study to document the lactation experience, breastfeeding outcomes, and milk composition for birthing parents with advanced CKD, kidney transplantation, or dialysis. Our primary objectives are to describe lactation outcomes (breastfeeding exclusivity, duration) and define milk composition (macronutrient, metabolome, electrolyte, cytokine, immunoglobulins) for this population. Secondary objectives include characterizing the breastfeeding experience (attitudes, self-efficacy, challenges) of birthing parents with CKD, kidney transplantation, or dialysis therapy and documenting the health outcomes of their infants. This work will contribute to the development of evidence-based and population-specific guidelines for breastfeeding among people with advanced CKD, kidney transplantation, dialysis therapy, and related conditions.

## Methods

### Research design

This is a prospective observational cohort study to describe lactation outcomes, breastfeeding experience, parent-infant health outcomes, and breast milk composition among birthing parents with advanced CKD, a history of kidney transplantation, or dialysis therapy (hemodialysis or peritoneal dialysis). Participants will be recruited during pregnancy or up to four weeks postpartum. Recruitment sites include the maternal-fetal medicine clinic, labor-delivery unit, mother-baby unit, and the kidney transplantation clinic at a tertiary academic medical center in Western United States as well as local dialysis clinics. In this study, we will not be assessing the gender identity of study participants. Therefore, we use the gender-inclusive term ‘birthing parent’ when describing our target population and ‘study participants’ when describing our cohort. An exception to this is instances when referencing work that uses gendered terminology.

**Fig. 1:**
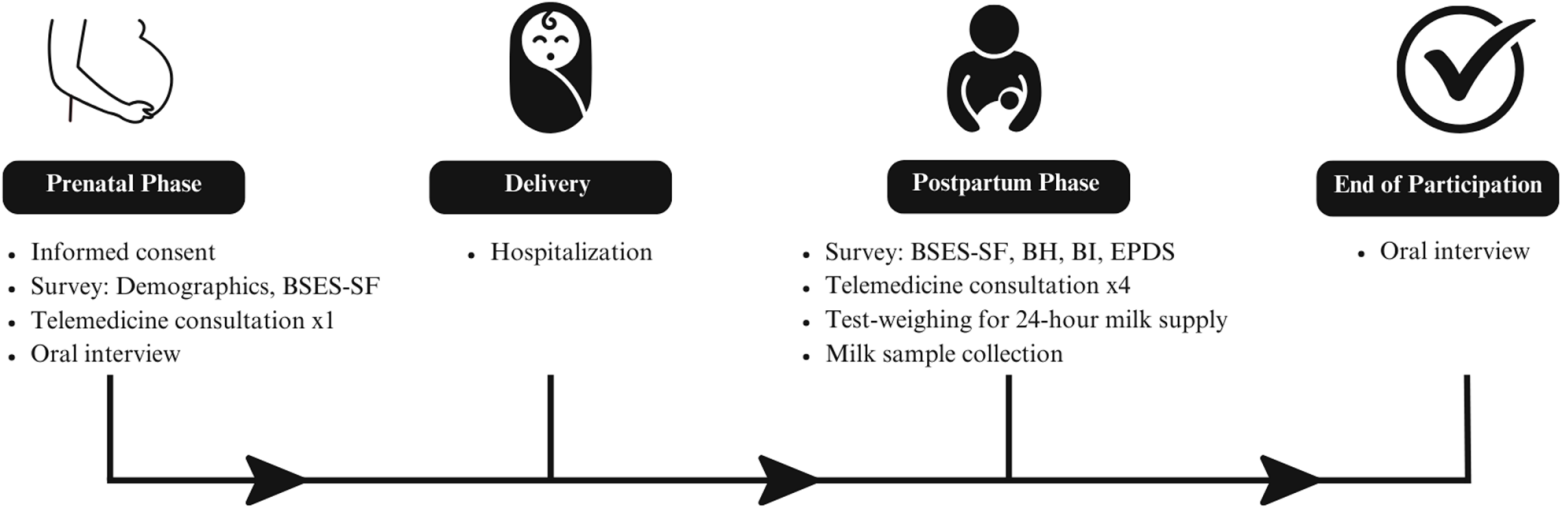
Study participation timeline. Overview of participant milestones from recruitment to the conclusion of participation in the study. Note BSES-SF= breastfeeding self-efficacy scale- short form, BH= breastfeeding history, BI= breastfeeding initiation, EPDS= Edinburgh Postnatal Depression Scale

For participants who are recruited prenatally, they will be asked to participate in the study starting during their third trimester of pregnancy and continuing until they are no longer breastfeeding or expressing milk, or the study concludes, whichever is first. Prenatally, participants will complete an oral interview, a breastfeeding telemedicine consultation, and a demographics and self-efficacy survey (Fig. 1). Postpartum, participants will have the opportunity to meet with a breastfeeding medicine specialist via telemedicine for up to four additional sessions. They will also complete self-efficacy, postpartum mood, and breastfeeding surveys, record their 24-hour milk supply, collect in-home milk samples, and participate in a final oral interview at the end of the study or lactation. Participants recruited postpartum will only complete the designated postpartum surveys. Participants will sign an informed consent document and HIPAA waiver to grant the investigator team access to their medical record, in addition to the infant’s chart.

The study integrates quantitative (e.g. surveys, chart reviews, and milk sampling) and qualitative (e.g. oral interviews and telemedicine consultations) methods to generate a comprehensive understanding of self-efficacy, breastfeeding attitudes and challenges, mood symptoms, parent-infant health outcomes, milk volume, and milk composition. Data will be collected and stored within secure software applications, including Research Electronic Data Capture (REDCap) and Microsoft SharePoint [21, 22]. The Institutional Review Board at our institution reviewed and approved the study protocol (2192067-2).

### Sample

We aim to recruit pregnant or postpartum Spanish- or English-speaking adults (*N* ~ 10) with advanced CKD Stage 4 or 5 (estimated glomerular filtration rate (eGFR) < 30 mL/min/1.73m^2^), receiving hemodialysis or peritoneal dialysis, or with a kidney transplantation (Table 1). On a case-by-case basis, pregnant patients with CKD stage 3b (eGFR 30–44 mL/min/1.73m^2^) will be considered. A board-certified nephrologist–the principal investigator for this study–will review the clinical history and laboratory data to evaluate the trends. Participants with rapidly progressing kidney disease will be included in the study and their laboratory data tracked throughout pregnancy and postpartum. Birthing parents who have true contraindications to breastfeeding will be excluded [23]. Participants will be compensated $300 as an e-gift card for their participation.

Healthy controls (*n* = 6) without kidney disease will be recruited from lactation support groups hosted at our institution. Based on historical data from birthing parents with CKD or metabolic disorders, we anticipate that most participants with CKD will produce low milk volumes (< 600 mL per day) and will be at a higher risk of early breastfeeding cessation by one month postpartum [11, 17]. We will recruit three control participants with low milk supply (< 600 ml) and three control participants with normal milk supply (600–1035 mL) between zero and eight weeks postpartum from the lactation support group. Controls will be compensated $100 as an e-gift card for their participation.

**Table 1:** Criteria for participant enrollment

Inclusion	Exclusion
≥ 18 years of age	Not interested in breastfeeding
Currently pregnant and planning to breastfeed **OR** Gave birth within the last month and breastfeeding	Birthing parent or infant contraindication to breastfeeding (e.g., HIV, galactosemia)
Advanced CKD (stage 4 or 5, or eGFR < 30 mL/min/1.73 m²)^a^ **OR** kidney transplantation recipient **OR** receiving dialysis^b^	eGFR > 44 mL/min/1.73 min^2^
	Non-English or Spanish speaking^c^

Note. eGFR = Estimated glomerular filtration rate

^a^On a case-by-case basis, CKD stage 3b (eGFR 30–44 mL/min/1.73 m²) will be considered for inclusion

^b^Hemodialysis or peritoneal dialysis

^c^Study team unable to offer telemedicine services in languages other than English or Spanish

### Data collection

Recruitment is expected to be completed within two years, along with data collection. Milk macronutrient composition will be batch analyzed every six months. Other milk components will be analyzed altogether in the final year of the study. Data analysis will be completed in year three.

### Survey instruments

A demographic survey instrument will be used to collect baseline information such as contact information, race and ethnicity, and breastfeeding experience and intention (Table 2). We will use three survey instruments (see the online Supplemental Material) to assess participants’ breastfeeding experience: Breastfeeding Initiation, Breastfeeding History, and the validated Breastfeeding Self-Efficacy Scale-Short Form (BSES-SF) [24–26]. The Breastfeeding Initiation survey, adapted from the Pregnancy Risk Assessment Monitoring System (PRAMS), evaluates participants’ experiences initiating breastfeeding in the hospital [27]. The Breastfeeding Initiation and History Survey instruments were designed by physician-scientists who specialize in breastfeeding medicine and are board-certified lactation consultants. The 24-item survey includes yes-or-no questions about participants’ interactions with the healthcare team during breastfeeding initiation in the hospital, the support they received, their infant feeding methods, whether pumping or formula supplies were provided, and when or if they experienced secretory activation. Since premature delivery is common in our study population, the Breastfeeding Initiation survey contains a branching logic question set that can be adapted for participants whose infant(s) are admitted to the NICU [11]. The Breastfeeding History survey, a 30-item survey, asks participants to recall their infant feeding practices and lactation experience over the prior two weeks, with questions about how the participant is feeding their infant, how often they are feeding their infant, how much milk they are producing, and any challenges while breastfeeding. The Breastfeeding History survey will also be administered to participants in the control group at the time of their milk sample collection. The validated 14-item BSES-SF will be used to assess breastfeeding self-efficacy [25]. We modified the verb tense in the BSES-SF survey for use prenatally (Prenatal BSES-SF). Symptoms of postpartum depression will be measured using the validated Edinburgh Postnatal Depression Scale (EPDS) [28] (Table 2). A plan has been developed to provide local mental health resources to study participants who score 12 or higher on the EPDS using their country-specific data.

**Table 2:** Survey administration timeline for participants with kidney disease

Survey	Prenatal	2 Weeks Postpartum	8 Weeks Postpartum	End of lactation or End of study
Demographics	✓			
Breastfeeding Self-Efficacy Scale	✓	✓	✓	✓
Breastfeeding Initiation		✓		
Breastfeeding History		✓	✓	✓
Edinburgh Postnatal Depression Scale		✓	✓	✓

#### Oral interview

Semi-structured, 30-minute oral interviews will be conducted prenatally and at the end of lactation or conclusion of the study, whichever is first. Interview prompts were informed from existing literature on breastfeeding barriers and experiences among birthing parents with chronic diseases [5, 11, 28]. See Supplemental Table 1 for prenatal and follow-up interview prompts. Interviews will be conducted through an institutional Zoom account, and audio recordings will be stored on the secure platform until they are transcribed and stored in a secured location (REDCap and Microsoft OneDrive).

#### Telemedicine consultation

Each participant will be offered five virtual consultations with a breastfeeding medicine physician using the institution’s telehealth platform - one prenatal and up to four postpartum. Consultations will be given at the following time points: third trimester, first 72 h postpartum, two weeks postpartum, between three and eight weeks postpartum, and one other time during the postpartum period if desired by the participant (Table 2). The time points were chosen based on key milestones during breastfeeding initiation and maintenance, aligning with national data on periods of postpartum breastfeeding challenges [6, 29]. Participants will be able to self-schedule using the breastfeeding medicine provider’s online calendar scheduler. Audio from the telehealth encounters will be transcribed within 30 days, and input as responses into REDCap.

#### Birthing parent and pediatric health history

When available, we will use the electronic medical record to collect data regarding birthing parents and infants’ health outcomes. Birthing parent data to be collected includes medical history, medications, pregnancy history, labor/delivery history, postpartum complications, and kidney disease progression, including the following relevant laboratory values: serum creatinine, eGFR, serum urea, electrolytes (sodium, potassium, phosphate, and calcium), and urinalysis variables when available and as appropriate, for example 24-hour urine protein or urine microalbumin to creatinine ratio. Pediatric data collected will include anthropometric measurements such as length, weight, and head circumference, growth chart percentiles, developmental milestones, and details of NICU admissions and relevant diagnoses, including chronic conditions. For study participants who are not part of the institution’s medical records, we will collect maternal and pediatric data using a survey instrument. Data will be recorded into a REDCap collection instrument [21, 22].

#### Milk volume and sample collection overview

All participants will be asked to measure their milk volume and provide at least one milk sample between three and eight weeks postpartum, when possible. Milk sample collection prior to solid food introduction will be prioritized. We will provide a milk sample collection kit and pediatric scale (Tanita BD-815U) to participants in-person at lactation support groups or clinic appointments. When required, we will ship the collection kit and scale to participants with a prepaid return label. All participants will be provided with detailed instructions of milk sample collection and shipment adapted from [30].

#### Measuring milk volume

Participants who are nursing will use a validated test-weighing technique to quantify milk supply once between three and eight weeks postpartum. Test-weighing involves placing the infant on a pediatric scale before and immediately after each nursing session on each breast and recording weight in grams. The difference between the pre- and post-nursing weight represents the amount of milk consumed by the infant (i.e. an estimate of breast milk supply) [17]. A milk log developed by the study team will enable participants to document infant weights during the test-weighing over a 24-hour period; this log will also allow the participant to record any milk that was expressed by hand or pump, as well as any formula or donor milk supplementation (see the online Supplemental Material). For participants on dialysis, additional fields will be used to document the start and end time of dialysis sessions, any breast symptoms experienced during or after dialysis, and whether breastfeeding or pumping occurred before or after dialysis. These details help contextualize milk volume data, particularly for birthing parents undergoing dialysis or managing complex comorbidities. This approach allows the study team to identify milk supply patterns, detect disruptions associated with treatment timing, and understand potential lactation barriers in medically complex populations [11, 18]. Study participants who are exclusively pumping will record expressed breast milk and any supplement provided to the infant over a 24-hour period. Instructions on test-weighing and using the milk log will be provided through a written document as well as a Zoom call with a member of the study team. If a study participant stops breastfeeding before we can ship the pediatric scale and collect milk samples, for example by 4 weeks postpartum, we will attempt to estimate 24-hour milk volume (low: <300 mL, moderately low: 300–600 mL, normal: 600–1035 mL, oversupply: >1035 mL) using participants self-reported milk supply, infant growth milestones, telemedicine consultations, and use of supplement from the Breastfeeding History survey.

#### Milk sample collection

**Fig. 2:**
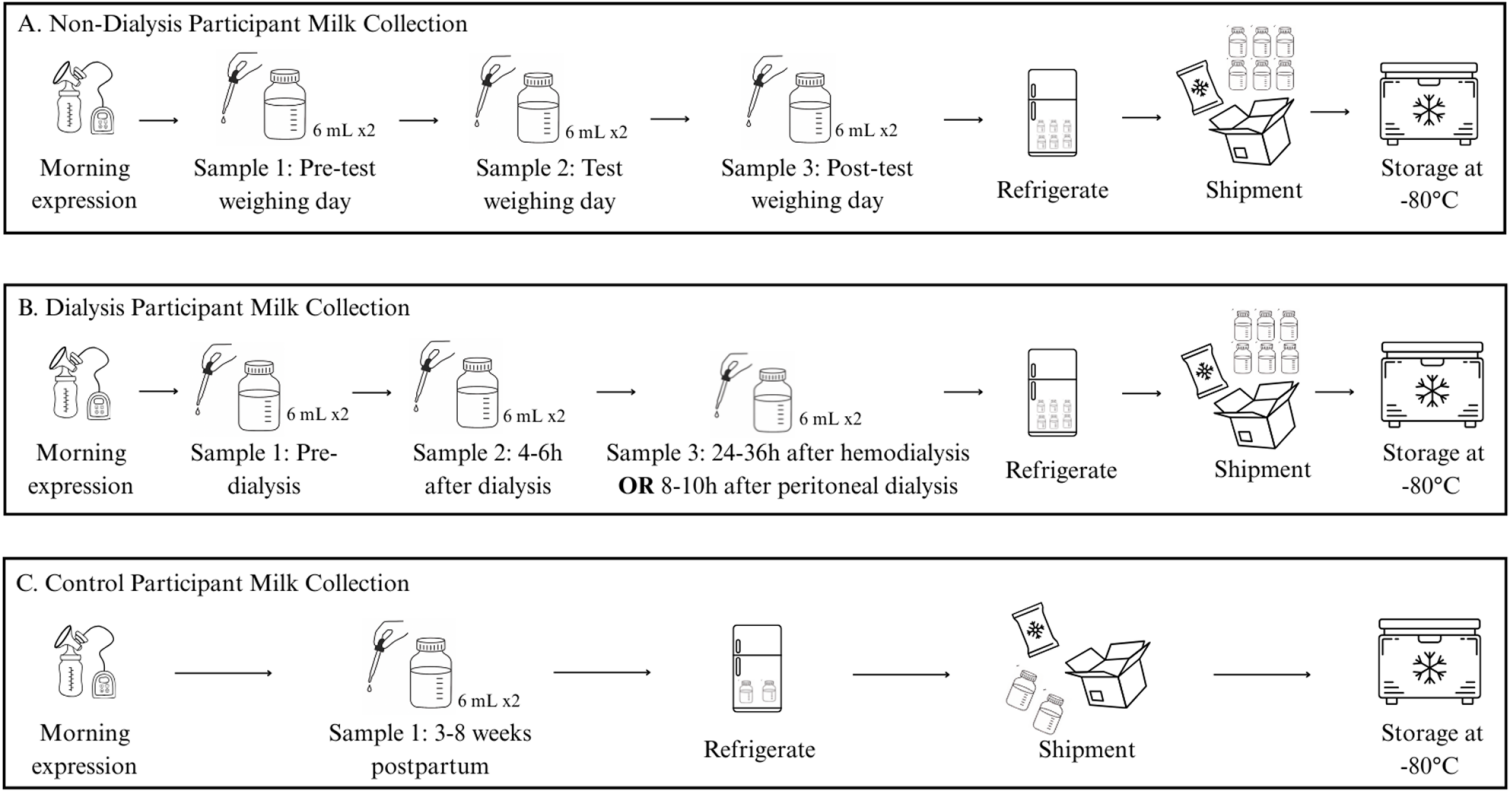
Milk sample collection. Milk sample collection procedures for non-dialysis, dialysis, and control participants

In the morning, participants will fully express one breast using a breast pump (Fig. 2). If participants have low milk supply and cannot express 12 mL from one breast in a single expression, they will be asked to express milk from both breasts and mix it together. Using a transfer pipette, they will place 6 mL of milk into one 15mL pre-labeled collection vessel and 6 mL into another. Participants with CKD or kidney transplantation will be asked to provide three milk samples (12 mL each split in two vessels) over three consecutive days, where the second day marks the 24-hour test-weighing period (Fig. 2A). For participants on dialysis, samples will be collected immediately before dialysis, 4–6 h after completing a dialysis treatment, and either 24–36 h after hemodialysis or 8–10 h after peritoneal dialysis (Fig. 2B) [12, 13]. Control participants will be asked to collect 12 mL of milk, equally split into two 6 mL samples in 15 mL collection vessels, from a morning expression between three and eight weeks postpartum (Fig. 2C). All participant samples will be stored in the refrigerator at home and shipped overnight with ice packs. Upon receipt in the NICU, one 6 mL sample will be frozen at -80 °C until batched macronutrient analysis, while the remainder will be frozen at -80 °C for future analyses.

### Outcomes/measurement

#### Primary outcomes

Our primary outcomes will be objective measures of breastfeeding exclusivity and duration, and a comparison of milk composition between groups, within groups, and over time (Table 3). We hypothesize that participants receiving dialysis therapy will have shorter breastfeeding duration compared to those with CKD or kidney transplantation [11–13]. We also hypothesize all participants to have equivalent or lower breastfeeding exclusivity rates compared to national averages [11]. Participants with advanced CKD are anticipated to have the highest pro-inflammatory milk, while those with a kidney transplantation are predicted to have lowest concentration of pro-inflammatory cytokines as a result of immunosuppressive medication [11–13, 19]. We expect that the level of inflammation in breast milk, as defined by cytokine content, will likely be comparable to that found in women with subclinical mastitis or other inflammatory diseases, like obesity or diabetes [31–33]. Birthing parents on dialysis will likely have the most variation in their milk electrolytes and macronutrient composition, with some values, such as high sodium and high protein, comparable to birthing parents with daily milk volumes less than 600 mL or those with subclinical or clinical mastitis [14, 31, 32, 34, 35].

**Table 3:** Details of study outcome measures

Outcome Measure	Measurement	Measure Detail
*Primary outcomes*		
Lactation outcomes	1. Breastfeeding initiation survey 2. Breastfeeding history survey	Breastfeeding initiation, exclusivity, and duration
Milk composition and volume	1. Test-weighing 2. 24-hour milk volume log 3. Milk sample(s) collection	Macronutrient, metabolome, electrolyte, cytokine, immunoglobulin content in milk; 24-hour milk volume
*Secondary outcomes*		
Breastfeeding experience	1) Oral interview 2) Telemedicine consultation 3) Breastfeeding Self Efficacy Scale survey 4) Breastfeeding History survey 5) Breastfeeding Initiation survey	Breastfeeding attitudes, self-efficacy, lactation challenges, support needs and concerns. Assessing feasibility of telemedicine consultations, participant satisfaction, and perceived impact on breastfeeding success and duration
Parent health outcomes	1. Edinburgh Postnatal Depression Scale 2. Parental medical record review	Correlation between postpartum mood and lactation outcomes, self-efficacy score, self-reported breastfeeding challenges, labor/delivery history, progression of kidney disease
Infant health outcomes	1. Pediatric health questionnaire 2. Pediatric medical record review	Hospitalization history, developmental milestones, medical history, and anthropometric data

#### Secondary outcomes

Our secondary outcomes are subjective descriptions of breastfeeding experiences, including breastfeeding attitudes, self-efficacy, lactation challenges, and objective measures of infant health. We expect that all participants will report a desire to breastfeed [11]. We also expect to find that birthing parents on dialysis, with advanced CKD (4 or 5), or with a kidney transplantation will have concerns ranging from medication compatibility to milk supply [11]. We hypothesize that other expected breastfeeding experience outcomes will likely align with previous breastfeeding literature, such as first time parents having low prenatal self-efficacy scores, parents with past history of low milk supply/breastfeeding difficulties will have low prenatal self-efficacy scores, and parents breastfeeding for the first time will be more likely to supplement due to perceived or real breast milk insufficiency [36–38]. We expect that a high EPDS score (> 12) will likely be associated with breastfeeding difficulties [39]. We hypothesize that participants will find the breastfeeding telemedicine encounters helpful and report that they felt their breastfeeding experience improved as a result of the advice they received from the consultations. For infant health outcomes, we expect that infants of birthing parents with CKD (4 or 5) or on dialysis will be more likely to be born premature (< 36 weeks) compared to infants of parents with CKD 3b or kidney transplantation [8–10]. Likewise, we hypothesize that infants of birthing parents with advanced CKD or on dialysis will be more likely to require a NICU stay [11]. When matched by gestational age at birth, we expect that infants will likely reach their developmental milestones appropriately, provided they do not have congenital or chronic illnesses.

### Planned analysis

#### Overview

Our study is not powered for inferential testing. Our mixed-methods analysis will be descriptive with the goal of hypothesis generation for future trials. Depending on the number of participants recruited in each category (e.g., advanced CKD versus kidney transplant), we will either present their data in aggregate or individually. Data from control groups will be presented in aggregate or separated by low (< 600 mL daily) and normal milk volume (≥ 600 mL daily), as appropriate. Triangulation of qualitative findings with quantitative results will allow for a more thorough understanding of the breastfeeding experience for birthing parents with advanced CKD, kidney transplantation or dialysis treatment, enabling the identification of both biologic and psychosocial drivers of lactation outcomes.

#### Quantitative data analysis

Descriptive statistics will be used to summarize demographic characteristics, lactation outcomes, participant medical history, labor and delivery outcomes, and infant medical history. Breastfeeding initiation, duration, exclusivity, and perceived and actual 24-hour milk volume will be compared between and within cases and controls. Categorical variables such as CKD stage, methods of feeding (e.g. nursing, pumping, both), milk volume (very low: < 300 mL, low: 300–599 mL, normal: 600–1050 mL, oversupply: > 1050 mL), and breastfeeding duration (none, 1, 2, 4, 6, 12 months, > 12 months) will be reported as percentages and frequencies and will be summarized using medians and interquartile ranges or means and standard deviations. Individual breastfeeding timelines may be plotted to explore variations in lactation patterns. Spearman Rank correlations will be used to assess the relationships between BSES and EPDS scores, BSES scores and breastfeeding outcomes, and EPDS scores and breastfeeding outcomes.

#### Qualitative data analysis

Qualitative data from telemedicine consultations and interviews will be analyzed using thematic analysis [40]. An inductive approach will be used, with codes developed from the data rather than predefined. Transcripts of the qualitative interviews and telemedicine consultations will be reviewed, and initial codes will be generated to capture key concepts related to breastfeeding decision-making, perceived barriers and facilitators, and the impact of kidney disease and treatment on lactation. Initial codes will subsequently be refined through repeated review across interviews, with codes merged, split, or redefined as needed to improve consistency and ensure representativeness. They will ultimately be organized into broader themes that reflect common experiences. Coding and theme development will be conducted by trained members of the study team, with regular discussions to address any discrepancies and ensure consistency. Qualitative findings will be integrated with quantitative measures (breastfeeding initiation, duration, and exclusivity, BSES scores, EPDS scores, and milk volume) to provide greater insight into the lactation experiences in this medically complex population.

#### Milk composition analysis

We will analyze macronutrients (total energy, lactose, protein, fat) using a Miris HMA machine. The milk metabolome, including mono-, di-, and oligosaccharides, amino acids and derivatives, energy metabolites, fatty acids and associated metabolites, vitamins, nucleotides, and derivatives, and other components will be analyzed using magnetic resonance spectroscopy methods described elsewhere [41]. Sodium and potassium content in milk will be assessed using ion-selective electrodes (Orion Ross, ThermoFisher Scientific). Over a dozen milk cytokines (HCYTA-60 K: IL-6, TNF-α, etc.) and immunoglobulins (HGAMMAG-301 K: IgG, sIgA, etc.) will be measured using multiplex bead assays (Millipore Sigma on Luminex Platform). Where appropriate, multivariate statistical techniques, such as principal component analysis, will be used to identify within- and between-group differences.

## Discussion

Pregnant women with advanced CKD, prior kidney transplantation, or on dialysis express a desire to breastfeed [11]. Many go on to breastfeed successfully for weeks or months postpartum, despite receiving conflicting advice from their healthcare team [11]. The inconsistency in advice is due to, in part, a paucity of high-quality data about the lactation experience and milk composition for this population. This study will highlight differences in milk composition compared to healthy controls and will shed light on the breastfeeding experience for birthing parents with advanced CKD, those on immunosuppressive medications post-kidney transplantation, or those receiving dialysis therapy. Through our mixed-methods approach, we aim to provide the multidisciplinary team of physicians, nurses, and allied health professionals who care for birthing parents and their infants with clear data on the barriers to successful breastfeeding, support needs for birthing parents with CKD, expected lactation outcomes, milk composition, and infant health outcomes. The data will help identify factors that result in early cessation of breastfeeding, enabling early intervention and the provision of evidence-based support.

### Limitations

Our tertiary healthcare center serves a diverse patient population, including birthing parents of various races, ethnicities, languages, and insurance status. One limitation of this study is that it will be conducted exclusively in English or Spanish, excluding other ethnic groups that reside within our catchment area, which may limit participant recruitment and the generalizability of the outcomes. Our anticipated sample size of 10 participants will not allow for inferential statistical analysis, which limits our ability to interpret our findings. Another limitation is the potential for user error due to sleep deprivation that comes with caring for a newborn during the 24-hour milk volume and sample collections. There is also a potential for recall bias or survey fatigue. These limitations will be addressed by spacing out survey administration and providing clear and simplified task instructions, with assurance and open communication around any lapses in documentation or recording.

### Protocol amendments

If changes are made to the study protocol, the IRB will be updated, and stakeholders will be notified.

## Supplementary Information

Below is the link to the electronic supplementary material.

Supplementary Material 1

Supplementary Material 2

Supplementary Material 3

Supplementary Material 4

Supplementary Material 5

Supplementary Material 6

## Data Availability

No datasets were generated or analysed during the current study.

## Abbreviations

BH: Breastfeeding history
BI: Breastfeeding initiation
BSES: SF-breastfeeding self-efficacy scale-short form
CKD: Chronic kidney disease
eGFR: estimated glomerular filtration rate
EPDS: Edinburgh postnatal depression scale
IRB: Institutional review board
NICU: Neonatal intensive care unit
REDCap: Research electronic data capture
