# Supplementary material for: Defining lactation outcomes, milk composition, and breastfeeding safety for women with chronic kidney disease: protocol for a prospective observational study
Source: Int Breastfeed J. 2026 Feb 21;21:36. doi: 10.1186/s13006-026-00821-0 (PMC13032694; doi:10.1186/s13006-026-00821-0)
Supplement: Supplementary file 1 — Supplementary Material 1 [file 13006_2026_821_MOESM1_ESM.pdf]

Breastfeeding History- 2 Month

Study ID ______________________

First name ______________________

Last name ______________________

Your baby's birth date ______________________

Section 1: How You Feed Your Baby

First, tell us how you want to measure milk
◯ Ounces (oz)
◯ Milliliters (mL)

How do you give milk to your baby now?

◯ Nursing at the breast only
◯ Pumping only
◯ Both nursing and pumping
◯ I no longer make milk
◯ Other

For "other," please describe

How much milk do you make each day?

◯ None (less than 1 oz per day)
◯ A small amount (1-10 oz per day)
◯ Some, but less than typical (11-23 oz per day)
◯ Typical amount (24-35 oz per day)
◯ More than typical (more than 35 oz per day)
◯ I'm not sure

How much milk do you make each day?

◯ None (less than 30 mL per day)
◯ A small amount (30-300 mL per day)
◯ Some, but less than typical (301-690 mL per day)
◯ Typical amount (691-1050 mL per day)
◯ More than typical (more than 1050 mL per day)
◯ I'm not sure

Do you take any pills or supplements to help make more milk?
◯ Yes
◯ No

Please list any pills or supplements that you take

Section 2: Nursing Your Baby

If you nurse your baby at the breast, how many times do you nurse in a day (24 hours)?

◯ I don't nurse my baby
◯ 1-3 times
◯ 4-7 times
◯ 8 or more times

Section 3: Pumping Your Milk

How many times do you pump in a day (24 hours)?

1 — 20

(Place a mark on the scale above)

How much milk do you usually get when you pump from both breasts?

1 — 20oz

(Place a mark on the scale above)

How much milk do you usually get when you pump from both breasts?

1 — 600mL

(Place a mark on the scale above)

How much milk do you pump total in a day (24 hours)?

1 — 100oz

(Place a mark on the scale above)

How much milk do you pump total in a day (24 hours)?

1 — 3000mL

(Place a mark on the scale above)

How much of your milk is in your freezer right now?

1 — 500oz

(Place a mark on the scale above)

How much of your milk is in your freezer right now?

1 — 1500mL

(Place a mark on the scale above)

In the past 2 weeks, what has your baby eaten? (Check all that apply)

☐ Only your breast milk (nursing or pumped)
☐ Formula
☐ Breast milk from another mother (donor milk)
☐ Solid foods
☐ Other drinks (water, juice, tea)

About what percent of their daily feeds are from other drinks such as water, juice, or tea?

1% — 100%

(Place a mark on the scale above)

About what percent of their daily feeds are from solid foods?

1% — 100%

(Place a mark on the scale above)

About what percent of their daily feeds are from donor milk?

1% — 100%

(Place a mark on the scale above)

About what percent of their daily feeds are formula?

1% — 100%

(Place a mark on the scale above)

Is there anything else you want to tell us about feeding your baby?

Section 5: Breast Problems or Concerns

Have you had any of these problems while feeding your baby in the past 2 weeks? (Check all that apply)

- ☐ Pain in your nipples
- ☐ Rash on your nipples
- ☐ White spots on your nipples
- ☐ Breast infection that needed antibiotics
- ☐ A painful lump in your breast that needed to be drained
- ☐ Lumps in your breasts
- ☐ Other problems
- ☐ No problems

For "other problems," please describe

Have your family members supported you with breastfeeding/pumping?

- ◯ Yes
- ◯ No

If no, please tell us why

Have your doctors and nurses supported you with breastfeeding/pumping?

- ◯ Yes
- ◯ No

If no, please tell us why
