# Supplementary material for: Defining lactation outcomes, milk composition, and breastfeeding safety for women with chronic kidney disease: protocol for a prospective observational study
Source: Int Breastfeed J. 2026 Feb 21;21:36. doi: 10.1186/s13006-026-00821-0 (PMC13032694; doi:10.1186/s13006-026-00821-0)
Supplement: Supplementary file 2 — Supplementary Material 2 [file 13006_2026_821_MOESM2_ESM.pdf]

Breastfeeding Initiation

Please complete the survey below.

Thank you!

First Name ______________________

Last Name ______________________

In the last two weeks, did you stop breastfeeding?
- ◯ Yes
- ◯ No

What were your reasons for stopping breastfeeding? (Check ALL that apply)

- ☐ My baby had difficulty latching or nursing
- ☐ Breast milk alone did not satisfy my baby
- ☐ I thought my baby was not gaining enough weight
- ☐ My nipples were sore, cracked, or bleeding
- ☐ It was too hard, painful, or too time consuming
- ☐ I thought I was not producing enough milk
- ☐ I had too many other household duties
- ☐ I felt it was the right time to stop breastfeeding
- ☐ I got sick or I had to stop for medical reasons
- ☐ I went back to work
- ☐ I went back to school
- ☐ My partner did not support breastfeeding
- ☐ Other

For "other," please describe

Did anyone suggest that you not breastfeed your new baby?
- ◯ Yes
- ◯ No

For "Yes," please describe

Was your baby hospitalized in the neonatal intensive care unit?
- ◯ Yes
- ◯ No

Did any of these occurrences take place at the hospital where your baby was born?

	No	Yes
Hospital staff gave me information about breastfeeding	○	○
My baby stayed in the same room with me at the hospital	○	○
I breastfed my baby in the hospital	○	○
Hospital staff helped me learn how to breastfeed	○	○
I breastfed in the first hour after my baby was born	○	○
My baby was fed only my breast milk at the hospital	○	○
Hospital staff told me to breastfeed whenever my baby wanted	○	○
The hospital gave me a breast pump to use	○	○
The hospital gave me a gift pack with formula	○	○
The hospital gave me a telephone number to call for help with breastfeeding	○	○
I felt my milk "come in" because my breasts became noticeably fuller.	○	○
My baby was fed formula in the hospital	○	○
My baby was fed water or sugar water in the hospital	○	○
My baby was fed donor human milk in the hospital	○	○

My breasts became noticeably fuller _______ .

○ Within a few days after I gave birth
○ By the end of the first week after I gave birth
○ Other

For "other," please describe.

Did any of these occurrences take place at the hospital where your baby was born?

	No	Yes
Hospital staff gave me information about breastfeeding	○	○
Hospital staff helped me learn how to breastfeed	○	○
I breastfed my baby in the NICU	○	○
I pumped while my baby was in the NICU	○	○
My baby was fed only my breast milk while in the NICU	○	○
The hospital gave me a breast pump to use	○	○
The hospital gave me a telephone number to call for help with breastfeeding	○	○
I felt my milk "come in" because my breasts became noticeably fuller	○	○
My baby was fed formula in the NICU	○	○
My baby was fed water or sugar water in the NICU	○	○
My baby was fed donor human milk in the NICU	○	○
