# Supplementary material for: Defining lactation outcomes, milk composition, and breastfeeding safety for women with chronic kidney disease: protocol for a prospective observational study
Source: Int Breastfeed J. 2026 Feb 21;21:36. doi: 10.1186/s13006-026-00821-0 (PMC13032694; doi:10.1186/s13006-026-00821-0)
Supplement: Supplementary file 3 — Supplementary Material 3 [file 13006_2026_821_MOESM3_ESM.pdf]

Breastfeeding Self-Efficacy Scale- 2 Week

Please complete the survey below.

Thank you!

1) First Name __________________________________

2) Last Name __________________________________

3) When did you give birth? __________________________________

For each of the following statements, please indicate how confident you are with breastfeeding by choosing the confidence level that most closely describes how you felt in the last two weeks.

In the last two weeks, I...

		Not at all confident	Not very confident	Sometimes confident	Confident	Very confident
4)	Could determine that my baby was getting enough milk	○	○	○	○	○
5)	Successfully coped with breastfeeding like I have with other challenging tasks	○	○	○	○	○
6)	Breastfed my baby without using formula as a supplement	○	○	○	○	○
7)	Could ensure that my baby was properly latched on for the whole feeding	○	○	○	○	○
8)	Managed the breastfeeding experience to my satisfaction	○	○	○	○	○
9)	Managed to breastfeed even if my baby was crying	○	○	○	○	○
10)	Continued wanting to breastfeed	○	○	○	○	○
11)	Comfortably breastfed with my family members present	○	○	○	○	○
12)	Was satisfied with my breastfeeding experience	○	○	○	○	○
13)	Could deal with the fact that breastfeeding can be time-consuming	○	○	○	○	○

14)

	Finished feeding my baby on one breast before switching to the other breast	○	○	○	○	○
15)	Continued to breastfeed my baby for every feeding	○	○	○	○	○
16)	Kept up with my baby's breastfeeding demands	○	○	○	○	○
17)	Could tell when my baby was finished breastfeeding	○	○	○	○	○
