# Supplementary material for: Defining lactation outcomes, milk composition, and breastfeeding safety for women with chronic kidney disease: protocol for a prospective observational study
Source: Int Breastfeed J. 2026 Feb 21;21:36. doi: 10.1186/s13006-026-00821-0 (PMC13032694; doi:10.1186/s13006-026-00821-0)
Supplement: Supplementary file 4 — Supplementary Material 4 [file 13006_2026_821_MOESM4_ESM.pdf]

Breastfeeding Self-Efficacy Scale- Prenatal

Please complete the survey below.

Thank you!

1) First Name ______________________________

2) Last Name ______________________________

3) When is your due date? ______________________________

For each of the following statements, please indicate how confident you feel about breastfeeding after delivery by choosing the response that most closely describes how you feel.

I am confident I will be able to...

		Not at all confident	Not very confident	Somewhat confident	Confident	Very confident
4)	Determine that my baby is getting enough milk	○	○	○	○	○
5)	Successfully cope with breastfeeding like I have with other challenging tasks	○	○	○	○	○
6)	Breastfeed my baby without using formula as a supplement	○	○	○	○	○
7)	Ensure that my baby is properly latched on for the whole feeding	○	○	○	○	○
8)	Manage the breastfeeding experience to my satisfaction	○	○	○	○	○
9)	Manage breastfeeding even if my baby is crying	○	○	○	○	○
10)	Continue wanting to breastfeed	○	○	○	○	○
11)	Comfortably breastfeed with my family members present	○	○	○	○	○
12)	Be satisfied with my breastfeeding experience	○	○	○	○	○
13)	Deal with the fact that breastfeeding can be time-consuming	○	○	○	○	○

14)

Finish feeding my baby on one breast before switching to the other breast	○	○	○	○	○
15) Continue to breastfeed my baby for every feeding	○	○	○	○	○
16) Keep up with my baby's breastfeeding demands	○	○	○	○	○
17) Tell when my baby is finished breastfeeding	○	○	○	○	○
