# Supplementary material for: Defining lactation outcomes, milk composition, and breastfeeding safety for women with chronic kidney disease: protocol for a prospective observational study
Source: Int Breastfeed J. 2026 Feb 21;21:36. doi: 10.1186/s13006-026-00821-0 (PMC13032694; doi:10.1186/s13006-026-00821-0)
Supplement: Supplementary file 5 — Supplementary Material 5 [file 13006_2026_821_MOESM5_ESM.pdf]

Milk log

Time *(circle AM or PM)*:	Right Breast		Left Breast		Donor milk or formula?		Notes
	**Before Feeding (g):**	**After Feeding (g):**	**Before Feeding (g):**	**After Feeding (g):**	**es/No *(circle Yes or No)*:**	**Total volume *(circle oz or mL)*:**	
AM / PM					Yes / No	oz / mL	
AM / PM					Yes / No	oz / mL	
AM / PM					Yes / No	oz / mL	
AM / PM					Yes / No	oz / mL	
AM / PM					Yes / No	oz / mL	
AM / PM					Yes / No	oz / mL	
AM / PM					Yes / No	oz / mL	
AM / PM					Yes / No	oz / mL	
AM / PM					Yes / No	oz / mL	
AM / PM					Yes / No	oz / mL	
AM / PM					Yes / No	oz / mL	
AM / PM					Yes / No	oz / mL	
AM / PM					Yes / No	oz / mL	
AM / PM					Yes / No	oz / mL	
AM / PM					Yes / No	oz / mL	
AM / PM					Yes / No	oz / mL	
AM / PM					Yes / No	oz / mL	
