# Supplementary material for: Defining lactation outcomes, milk composition, and breastfeeding safety for women with chronic kidney disease: protocol for a prospective observational study
Source: Int Breastfeed J. 2026 Feb 21;21:36. doi: 10.1186/s13006-026-00821-0 (PMC13032694; doi:10.1186/s13006-026-00821-0)
Supplement: Supplementary file 6 — Supplementary Material 6 [file 13006_2026_821_MOESM6_ESM.pdf]

Table S1. Qualitative Interview Guide

Section	Interview Prompt & Questions
**A. Prenatal Interview Guide (During Pregnancy/Recruitment)**	
Introduction Script	“Is it OK for me to start recording? <<Press record>> Thank you for participating in this interview. We're interested in learning about your thoughts on infant feeding as someone with kidney disease. There are no right or wrong answers—we want to understand your plans, hopes, and concerns. Please feel free to share as much or as little as you're comfortable with.”
Current Situation & Feeding Plans	1. Could you tell me about your plans for feeding your baby?<br>• What has influenced your thinking?<br>• If prior breastfeeding experience:<br>○ Tell me more about your previous breastfeeding experience.<br>○ Duration of breastfeeding?<br>○ Difficulties with milk supply?<br>○ Did doctors support breastfeeding? Why/why not?<br>○ Did kidney disease or treatment interfere?<br>• How do you feel about these plans?
Breastfeeding Concerns	2. What concerns do you have about breastfeeding with kidney disease?<br>• What worries you most?<br>• How have you been thinking about managing these concerns?
Healthcare Support & Preparation	3. What discussions have you had with your healthcare team about breastfeeding?<br>• What information have different providers given you?<br>• What additional information would be helpful?<br><br>4. What discussions have you had about medications and breastfeeding?<br>• Concerns about medications?

	• Clarity on medication safety?<br><br>*If on dialysis:<br><br>• Discussions about dialysis and breastfeeding?<br>• Concerns? Clarity on dialysis safety?
Support Planning	5. How are you planning to manage breastfeeding with your kidney care routine?<br>• Anticipated challenges?<br>• Strategies?<br><br>6. What support systems do you have in place after birth?<br>• Who will be helping?<br>• Additional support needed?
**B. Follow-Up Interview Guide (End of Lactation / Study Period)**	
Introduction Script	"Thank you for speaking with us again. We'd like to learn about your actual experiences with infant feeding. Please be candid about what worked well and what didn't."
Infant Feeding Experience	1. Could you tell me how your infant feeding experience has gone?<br>• Comparison to expectations?<br>• Surprises?
Impact of Kidney Disease	2. How has your kidney condition affected your breastfeeding journey?<br>• Challenges?<br>• How did you address these challenges?
Healthcare Support Experience	3. How would you describe the support you received from your healthcare team?<br>• Most helpful?<br>• Support that was missing?<br><br>4. Experience with telemedicine lactation consultations?<br>• Helpfulness?<br>• What could have improved them?
Practical Management	5. How did you manage breastfeeding with your kidney care routine?<br>• Strategies that worked?

	• Most challenging aspects?<br><br>6. Medications while breastfeeding?<br>• How were decisions made?<br>• Concerns?
Support Systems	7. Who or what provided the most valuable support during breastfeeding?<br>• What made this support helpful?<br>• What additional support would've helped?
Reflection & Recommendations	8. Advice for other women with kidney disease planning to breastfeed?<br><br>9. Recommended changes to better support these women?<br><br>10. Anything else to share?
Probing Questions<br><br>(Used as needed throughout)	• Could you tell me more about that?<br>• How did that make you feel?<br>• Could you give an example?<br>• What do you mean by…?<br>• How did you handle that situation?
